# Gene silencing of HPV16 E6/E7 induced by promoter-targeting siRNA in SiHa cells

**DOI:** 10.1038/sj.bjc.6605344

**Published:** 2009-10-13

**Authors:** D Hong, W Lu, F Ye, Y Hu, X Xie

**Affiliations:** 1 Women's Reproductive Health Laboratory of Zhejiang Province and Department of Gynecologic Oncology, Women's Hospital, School of Medicine, Zhejiang University, Hangzhou 310006, China

**Keywords:** small interfering RNA (siRNA), human papillomavirus (HPV), transcriptional gene silencing (TGS), senescence, histone methylation

## Abstract

**Background:**

Recently, transcriptional gene silencing induced by small interfering RNA (siRNA) was found in mammalian and human cells. However, previous studies focused on endogenous genes.

**Methods:**

In this study, we designed siRNA targeting the promoter of human papillomavirus 16 E6/E7 and transfected it into the cervical cancer cell line, SiHa. E6 and E7 mRNA and protein expression were detected in cells treated by promoter-targeting siRNA. Futhermore, cellular growth, proliferation, apoptosis and senescence were detected. Thereafter, we investigated promoter DNA methylation and histone methylation status in cells treated with promoter-targeting siRNA.

**Results:**

We found that E6/E7 mRNA and protein were simultaneously reduced, cell growth and proliferation were inhibited and cell death, especially senescence, was remarkably increased. Meanwhile, we also found a significantly increasing histone H3-Lys9 methylation on the promoter when E6/E7 gene expression was inhibited.

**Interpretation:**

Our findings suggest that promoter-targeting siRNA effectively and simultaneously knocks down both extraneous HPV 16 E6 and E7 at the transcriptional level, and consequently inhibits proliferation and induces death in HPV 16-infected cells. This transcriptional repression is probably induced by histone modification rather than by alteration of DNA methylation.

## Background

Small interfering RNA (siRNA) has been widely used to degrade homologous mRNA at the post-transcriptional level. Recent studies found that siRNA also targeted DNA and induced transcriptional gene silencing (TGS). Initially, siRNA-mediated TGS was observed in plants through the use of inverted repeat transgenes to generate siRNAs homologous to the target promoter (Mette *et al*, 2000). This kind of strategy has been demonstrated to be useful for transcriptionally downregulating genes in a variety of plants (Jones *et al*, 2001; Sijen *et al*, 2001). Recently, transcriptional gene silencing induced by siRNA was also found in mammalian cells (Morris *et al*, 2004; Ting *et al*, 2005). It was reported that a siRNA, which was designed homologous to an elongation factor 1 alpha (EF1A) promoter sequence and transfected with an FIV vector, induced downstream gene silencing at the transcriptional level in human 293FT cells (Morris *et al*, 2004). In addition, a synthetic siRNA targeted exclusively to the CDH1 promoter could effectively induce transcriptional repression with chromatin changes characteristic of inactive promoters. Those results indicate that siRNA-induced TGS offers an additional way of silencing genes, other than posttranscriptional gene silencing (PTGS). However, most of the previous studies focused on endogenous genes, especially on some inactivation mechanism of tumour-suppressor genes. It has not been known till date whether a promoter-targeting siRNA could induce gene silencing of an extraneous viral gene such as human papillomavirus (HPV).

Human papillomavirus is a common type of DNA virus that infects humans. Of them, high-risk HPV is associated with the occurrence of cervical carcinoma. In a worldwide analysis, the prevalence of high-risk HPV in cervical cancer was 99.7%, and persistent infection of HPV is recognised to be a necessary cause of cervical carcinoma (Walboomers *et al*, 1999). In addition, high-risk HPV is also found to be a pathogenic factor associated with other cancers, such as vulva, vaginal, rectal and oral cancer (Miller and Johnstone, 2001; Daling *et al*, 2004). Among all the HPV genotypes, HPV 16 is the most prevalent, with a proportion of 65.2% of all genotypes in cervical cancer (Hong *et al*, 2008). Various publications have demonstrated that continuous expression of both viral oncogenes, E6 and E7, is necessary during HPV induction of malignant transformation and maintenance in cervical epithelial cells (Goodwin and DiMaio, 2000; Munger *et al*, 2004). Considering the crucial effect of E6/E7 in carcinogenesis, almost all studies focus on the induction of PTGS of E6 and/or E7 expression (Jiang and Milner, 2002; Yoshinouchi *et al*, 2003). It has been found that the expression of HPV 16 E6 and E7 is controlled by a common promoter (P97) that is located immediately upstream of the E6 gene (Smotkin and Wettstein, 1986). The promoter region contains several binding sites for cellular transcription factors such as TFIID (Dostatni *et al*, 1991), Sp1 and E2 (Tan *et al*, 1992), all of which modulate its function. The modification of DNA or histones in the region will influence the function of the promoter. For example, methylation within the binding sites of transcription factors such as Sp1 might block binding indirectly, either by changing the conformation of chromatin or by interacting with methyl-CpG-specific repressor proteins (Zhu *et al*, 2003). It is generally believed that the strength of transcription from P97 will determine the concentration of the oncoprotein E6/E7 (Stunkel *et al*, 2000). The common promoter of E6/E7 offers a possibility to knock down the E6/E7 gene simultaneously by means of siRNA-mediated TGS.

In this study, we designed siRNA targeting the promoter of E6/E7 to explore whether it is likely to knock down both genes simultaneously at the transcriptional level, and consequently inhibit proliferation and induce death in HPV-positive cells. After E6/E7 silencing was observed, we further investigated its association with promoter DNA methylation and histone methylation to explore a novel mechanism of HPV E6/E7 silencing by siRNA.

## Methods

### Design and synthesis of promoter-targeting siRNA

The criteria described by Sohail *et al* (2003) and the computer programme available at http://www.promega.com/siRNADesigner/program/ were used to design siRNA. The T7 RiboMAX Express RNAi System Kit (Promega, Madison, WI, USA) was used to synthesise siRNA. The sequence of siRNA was designed to complement the common promoter P97 of E6/E7 in HPV 16 (NC_001526). The promoter-targeting siRNA contained 21 nucleotides (nt) of the target sequence, homologous to the promoter of E6/E7 (position in gene sequence 32–52 nt). The promoter-targeting siRNA sense strand was GUAACCGAAAUCGGUUGAACC and the promoter-targeting siRNA antisense strand was CAUUGGCUUUAGCCAACUUGG. A scrambled siRNA was used as negative control. The sequence was submitted to a BLAST search against the human genome sequence to ensure that no gene of the human genome was targeted.

### Cell culture and siRNA transfection

A human cervical carcinoma cell line, SiHa, was used in this study. In SiHa cells, HPV16 DNA is integrated into the cellular genome. Moreover, DNA in this region is unmethylated.

Three groups were included in the experiments: the promoter-targeting siRNA transfected with liposome was considered as the experiment group; scrambled siRNA transfected with liposome was considered as negative control and liposome without siRNA as blank control.

SiHa cells were transferred to 12-well plates for most experiments. However, 6-well and 96-well plates, and 60-mm dishes were used in apoptosis, proliferation and histone detection experiment according to the introduction of the kit. The dose of siRNA used was altered correspondingly.

SiHa cells were transferred to the plate and cultured for 24 h. An appropriate cell density of 30–40% was chosen before siRNA transfection. siRNA was transferred with the liposome of CodeBreaker siRNA Transfection Reagent (Promega, WI, USA) according to the manufacturer's instructions. After 24 h of incubation, the cells were washed once, and after 48 h, they were collected.

Before the experiment, we used siRNAs in SiHa with different concentrations (20, 40, 60 and 80 *μ*M). Finally, we selected 40 *μ*M siRNA as the experiment concentration, according to the growth state of the negative control and the mRNA expression level of E6 and E7 in SiHa transferred with promoter-targeting siRNA (data not shown). Data were collected from three trials of the same experiment.

### Detection of E6 and E7 expression

E6 E7 mRNA was detected by RT–PCR. The amplified regions were both in the exons of E6 (position in gene sequence 421–540 nt) and E7 genes (642–774 nt). Total RNA was obtained from cells with Trizol and chloroform. After RNA identification and DNA digestion, mRNA was reversely transcripted to single-stranded cDNA with oligodT (18). cDNA samples were diluted and subjected to PCR amplification with forward and reverse primers specific to HPV16 E6, E7 and *β*-actin (seen in Table 1). PCR analyses of E6 and E7 cDNA fragments were performed under the following conditions: denaturation was at 94°C for 5 min, followed by 24–26 PCR cycles to ensure amplification in the linear stage (denaturation at 94°C for 30 s, primer annealing at 58°C for 1 min, primer extension at 68°C for 2 min). *β*-Actin cDNA was detected under almost the same conditions, with three cycles fewer. For every PCR assay, a negative control and a positive control were used to avoid possible false positives and false negatives. Amplified products were separated on 2% agarose gels. Intensities of E6 and E7 bands were quantified using the Image Gauge software and normalised to those of *β*-actin bands.

Because E6 proteins were unsuccessfully detected by western blotting with several commercial anti-E6 antibodies (Butz *et al*, 2003), E6 and E7 proteins were only detected by immunocytochemistry. Cells were seeded on slides in 12-well plates. After siRNA was treated for 48 h, 4% paraformaldehyde was used to fix cells on the slides and 0.1% Triton X-100 was used to break the nuclear member. Subsequently, slides were incubated with monoclonal antibodies to E6 (Calbiochem, Darmstadt, Germany) diluted 1 : 50 and to E7 (Santa Cruz, Santa Cruz, CA, USA) diluted 1 : 75 separately in PBS for 1 h, and then incubated with Dako Envision peroxidase for 1 h. Thereafter, 3,3′-diaminobenzidine tetrahydrochloride was added for visualisation and Mayer's haematoxylin was used to counterstaining. Positive cells were indicated by the presence of a distinct brown stain in the nucleus or cytoplasm of cells. Treatment of blank control was performed by replacing E6 or E7 antibodies with normal rabbit serum. Treatment of negative control was performed using C33A (a cervical cancer cell without HPV infection). The immunoreaction of E6 and E7 protein expression was semi-quantitatively scored in the same manner as that in previous study (Innocenzi *et al*, 2003).

### Detection of cellular growth, proliferation, apoptosis and senescence

Cellular growth, including density and morphology of cells, was observed using a phase-contrast microscope. On the basis of the measurement of BrdU incorporation during DNA synthesis, cellular proliferation was detected by colorimetric immunoassay using a Cell Proliferation ELISA, BrdU (colorimetric) kit (Roche) according to the manufacturer's instructions. The reaction product was quantified by measuring absorbance at the respective wavelength using a scanning spectrophotometer. The absorbance values were directly correlated to the amount of DNA synthesis and, thereby, to the number of proliferating cells.

To assess the rate of cell apoptosis, annexin-V binding and propidium iodide permeability were examined by flow cytometry. Apoptosis cells were quantified by annexin-V–FITC and propidium iodide double staining using an Annexin-V/FITC kit (Bender MedSystems, Burlingame, CA, USA), according to the manufacturer's instructions. The log of annexin-V–FITC and propidium iodide fluorescence was displayed on the *x*- and *y*-axis of the cytogram, respectively. Annexin-V-positive cells plotted in the lower and upper right quadrants were evaluated as dead cells.

Senescence-associated *β*-galactosidase assay (SA*β*-gal) was used to detect cell senescence using a cell senescence testing kit (Genmed Scientifics, Arlington, VA, USA). After treatment with siRNA for 48 h, SiHa cells were fixed and incubated overnight with an SA*β*-gal staining solution at 37°C until a green precipitate appeared. The positive cells were counted by light microscopy at × 200 magnification and five random fields were also counted.

### Detection of DNA methylation

Different methods, including Sanger DNA sequencing, methylation-specific PCR and pyrosequencing, were used to detect DNA methylation in the promoter of E6 and E7. All these methods were based on PCR and the template was treated with sodium bisulphite. Bisulphite treatment of DNA converted epigenetic information into a genetic polymorphism. Genomic DNA was treated with sodium bisulphite as before (Grunau *et al*, 2001) to transfer unmethylated C to T, and to maintain methylated C as C. All primer sequences are shown in Table 1.

First, Sanger DNA sequencing of PCR amplifier was performed. Semi-nested PCR was used to acquire the amplifier. The outer primers of PCR were msp7708F and msp161R (319 bp), and the inner primers were msp7708F and msp115R (273 bp). If the CpG sites in the promoter became methylated, two apices of C and T would simultaneously show in the CpG position.

Thereafter, methylation-specific PCR (MSP) was used when sequencing did not differentiate between the methylated and unmethylated state. MSP distinguishes between unmethylated and methylated alleles by using two sets of primers that amplify either unmethylated or methylated sequences after bisulphite treatment. In this study, we used a pre-amplified common PCR product as the template of MSP for sensitivity of detection. The outer PCR used common primers of msp7708F and msp161R, and the inner PCR used a methylation primer and an unmethylation primer. The methylation primers used in this study were msp7708F and 53mR, and the unmethylation primers were msp7708F and 53 nmR.

Finally, a quantifiable method of pyrosequencing was used to verify the result of MSP. Pyrosequencing is a good method for analysing methylation status with simultaneous quantification (Dupont *et al*, 2004; Wong *et al*, 2006). The accuracy of pyrosequencing is not less than that of subcloning and sequencing. The advantage of the method was to quantify the percentage of C and T in the target CpG site. The method was also based on PCR. The primers used in the PCR were approximately identical with those for Sanger DNA sequencing, with only the reverse primer 115R biotinylated. The primer of pyrosequencing was pyro-msp11F. The protocol was as usual (Rajeevan *et al*, 2006).

### Detection of histone methylation

We detected trimethyl-histone H3-Lys9 (H3K9me3) by chromatin immunoprecipitation (ChIP) assay and real-time PCR. We processed SiHa cells in accordance with the Upstate ChIP assay kit protocol (17-611, Upstate, Temecula, CA, USA), and used ChIPAb+trimethyl-histone H3 (Lys9) (17-625, Temecula, CA, USA, Upstate) as the special antibody. We analysed the ChIP results by real-time PCR using SYBR Premix Ex Taq (Takara, Dalian, China) with a set of HPV 16 promoter-specific primers (F16 and R 113) spanning the HPV 16 promoter (position in gene sequence 16-113nt). We also performed PCR of GAPDH (GAPDH-F and GAPDH-R) on the same sets of immunoprecipitated DNA fractions as controls.

### Statistical analysis

Data were analysed with independent-samples *t-*test and the Mann–Whitney test using SPSS version 11.5 for Windows. All *P*-values less than 0.05 were considered statistically significant. All reported *P*-values were bilateral.

## Results

### Simultaneously reduced expression of the E6 and E7 oncogene by siRNA targeting the E6/E7 promoter

**Figure 1:**
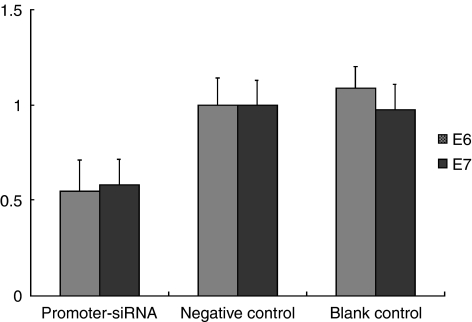
The expression of HPV16 E6 and E7 mRNA in SiHa cells by promoter–siRNA. E6 and E7 mRNA expression detected by RT–PCR. The *x*-axes show different experiment groups (promoter siRNA, negative control, blank control); *y*-axes show the E6/E7 level, which was normalised to that of negative control. E6 and E7 mRNA expression was decreased by 45.2 and 41.7% separately as compared with the negative control.

HPV 16 E6 and E7 mRNA expressions were determined by semi-quantitative RT–PCR. In SiHa cells transfected by promoter-targeting siRNA, the E6 mRNA expression was decreased by 45.2% as compared with that in the negative control (*P*=0.022), and decreased by 49.7% as compared with that in the blank control (*P*=0.009). At the same time, E7 mRNA expression was simultaneously decreased by 41.7 and 40.2%, respectively, as compared with that in the negative control (*P*=0.017) and blank control (*P*=0.022; Figure 1).

**Figure 2:**
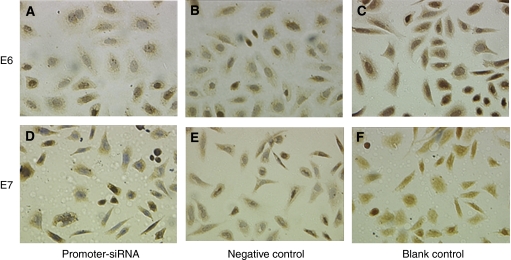
The expression of HPV16 E6 and E7 proteins in SiHa cells by promoter–siRNA. E6 and E7 protein expression detected by immunocytochemistry (400 × ). E6 protein (**A**–**C**) and E7 protein (**D**–**F**) were mainly located in the nucleus. If the protein is highly expressed, the nucleus and plasma were stained brown. The percentage of cell stained was measured by image analysis from 10 random fields from each cell slide, and data were pooled to determine the mean. It was shown that E6 and E7 proteins in SiHa treated with promoter–siRNA (A, D) were expressed lower than that of the negative control (B, E) and blank control (C, F).

The expression of E6 and E7 protein in cells was determined by immunocytochemistry. Semi-quantitative analysis showed that E6 protein expression in SiHa cells treated with promoter-targeting siRNA was decreased by 33.3 and 37.1%, respectively, as compared with that in the negative control (*P*=0.009) and blank control (*P*=0.002). Simultaneously, the E7 protein expression in SiHa cells treated with promoter-targeting siRNA was decreased by 30.3 and 34.2%, respectively, as compared with that in the negative control (*P*=0.017) and blank control (*P*=0.004; Figure 2).

### Reduced cellular growth and proliferation, increased apoptosis and senescence in SiHa cells treated by promoter-siRNA

**Figure 3:**
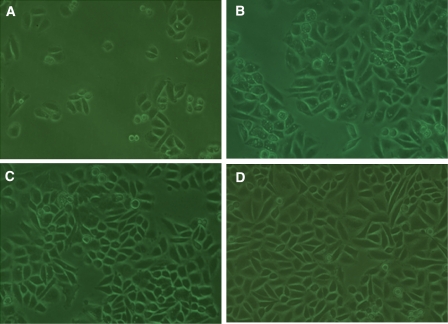
The cellular growth aspect of SiHa cells treated by promoter–siRNA. (**A**) SiHa before transfection (0 h). (**B**) SiHa treated with promoter-targeting siRNA (48 h), the dark arrows point to cells with vacuoles. (**C**) Negative control: SiHa treated with scrambled siRNA (48 h). (**D**) Blank control: SiHa treated without siRNA (48 h). The promoter-targeting siRNA-treated cells (**B**) showed slower growth, with some small vacuoles in the cytoplasm of cells.

To examine whether suppression of E6 and E7 affected cell growth, cellular morphology, proliferation, apoptosis and senescence were detected. At 48 h after transfection, cell morphology was changed in promoter-targeting siRNA-treated cells as compared with that in the negative and blank control. Promoter-targeting siRNA-treated cells showed slower growth, with some small vacuoles in the cytoplasm of cells, but no morphological change was found in the negative and blank control cells (Figure 3).

**Figure 4:**
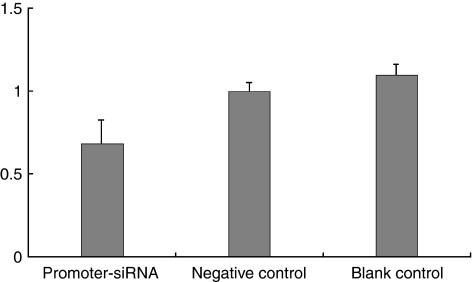
Cellular proliferation in SiHa cells treated by promoter–siRNA. Proliferation rates of SiHa cells treated by siRNA. The *x*-axes show different experiment groups and *y*-axes show cell proliferation rates, which were normalised to that of the negative control.

The cellular proliferation state was also detected by colorimetric immunoassay with Cell Proliferation ELISA of BrdU. For cellular proliferative rate, cells treated by promoter-targeting siRNA showed a 22.2 and 29.8% decrease than the negative (*P*=0.000) and blank control (*P*=0.000; Figure 4).

**Figure 5:**
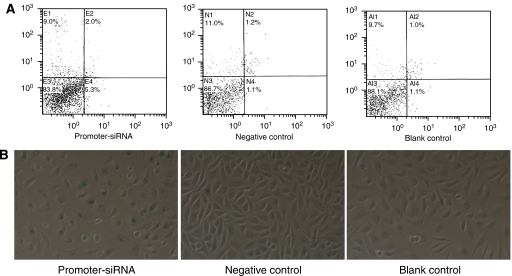
Cellular apoptosis and senescence in SiHa cells treated by promoter–siRNA. (**A**). Apoptosis of SiHa cells treated by siRNA. In the flow cytometry chart, the *x*-axes represent the fluorescence intention of annexin-V–FITC, and *y*-axes show the fluorescence intensity of propidium iodide and the dot in the quadrant represents the cell number. The right down quadrant shows apoptosis data. (**B**) Senescence of SiHa cells treated by siRNA (200 × ). Cells that showed intense green staining reflected high levels of SA*β*-gal activity. Further green staining appeared in cells treated with promoter-targeting siRNA. The colour version of this figure is available online.

Apoptotic cells were quantified by annexin-V–FITC by flow cytometry. Generally, the apoptotic rates were relatively low. The apoptotic rate was 4.4±0.8, 1.7±1.0 and 1.6±0.6%, respectively, for cell treated with promoter-targeting siRNA, the negative control and blank control. However, the apoptotic cells in the experiment group were also more than those in the negative (*P*=0.009) and blank control (*P*=0.022; Figure 5A).

Senescence-associated *β*-galactosidase assay was used to detect cell senescence. The senescence rate of cells treated by promoter-targeting siRNA was 57.1±4.4%, which was significantly higher than that of the negative control (35.8±3.1%, *P*=0.000) and blank control (32.8±4.0%, *P*=0.000; Figure 5B).

### No DNA methylation alteration but increased H3K9me3 in the promoter of HPV16 in SiHa cells treated by promoter-siRNA

**Figure 6:**
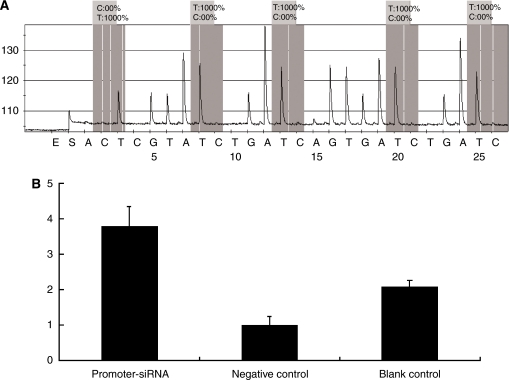
Detection of DNA methylation and H3K9 methylation of the HPV 16 E6/E7 promoter in SiHa cells treated by siRNA. (**A**) Sequence of pyrosequencing of the E6/E7 promoter. Genomic DNA was treated with sodium bisulphite to transfer unmethylated C to T, and to maintain methylated C as C. Therefore, in the results of pyrosequencing, T represents unmethylated C. Five CpGs in the promoter region were all unmethylated. (**B**) H3K9me3 expression detected by ChIP and real-time PCR. *ΔΔ*C_(t)_ were 0.86±0.13, 0.23±0.06 and 0.48±0.04 separately in the promoter–siRNA, negative control and blank control. The *x*-axes show different experiment groups, and *y*-axes show H3K9me3 expression, which was normalised to that of the negative control.

Although MSP analysis showed mixed methylation in promoter-targeting siRNA-treated cells, the result could not be verified in both Sanger DNA sequencing and pyrosequencing. Sanger DNA sequencing showed a single peak of T in the sequence map, indicating that no methylation was detected. Pyrosequening showed that the frequency of T (represents the unmethylated G) was 100% in all of the five CpG sites of the HPV16 promoter (Figure 6A).

Chromatin immunoprecipitation assay and real-time PCR showed that H3K9me3 adjacent to the promoter was significantly increased in promoter-targeting siRNA-treated cells than in both the controls (Figure 6B).

### Interpretation

Previous studies demonstrated that the siRNA-targeting promoter DNA can induce downstream gene silencing at the transcriptional level in human and mammary cells. In this study, we found that siRNA targeting the promoter of E6/E7 effectively and simultaneously inhibited the expression of both E6 and E7 in SiHa cells. At the same time, our data confirmed that the activity of siRNA-targeting promoter was at the transcriptional level. To our knowledge, it is the first report that a promoter-targeting siRNA induces the silence of extrinsic viral genes of HPV. Our study showed that siRNA-targeting promoter interferes with mRNA transcription, reaching more than 40 and 30%, respectively decrease in E6/E7 mRNA and protein, respectively, suggesting that the siRNA-targeting promoter of E6/E7 is able to effectively knock down E6/E7 expression simultaneously.

A unique feature of HPV-associated cancer is that the maintenance of the malignant aspect and behaviour of cancer cells is dependent on the expression of viral protein E6 and E7. In case the E6/E7 expression is inhibited, the cellular malignant characteristics would be abrogated. In our study, the moderate decreases in E6/E7 mRNA and protein expression also partially altered the malignant characteristics. At the same time, cells treated by the siRNA-targeting E6/E7 promoter represented a remarkably decreased proliferation, as well as changed cellular growth morphology. Furthermore, we found that cell death, including apoptosis and senescence, was induced; it is to be especially noted that senescence was remarkably increased when E6 and E7 expression was inhibited at the transcriptional level. It is interesting that senescence is a more common form than apoptosis in cell death during promoter-targeting siRNA inducing transcriptional gene silencing (Butz *et al*, 2003; Hall and Alexander, 2003; Horner *et al*, 2004). E7 acts as a mitotic mutator and growth-stimulatory factor that interferes with cellular senescence and proper differentiation, whereas E6 counteracts the apoptotic response of HPV-positive cells towards the abnormal growth stimulus exerted by E7, ultimately resulting in deregulated growth of genetically unstable cells (Mantovani and Banks, 2001). Thus, when E6 alone is targeted, cells readily become apoptotic. However, when both are inhibited, senescence is predominantly droven and apoptosis is reduced. (Butz *et al*, 2003; Hall and Alexander, 2003).

Recent studies of mammalian cells indicate that siRNA-mediated transcriptional gene silencing is likely to be affected by multiple epigenetic factors, such as histone modification and cytosine methylation. There are some data that a histone-methylation-mediated heterochromatin pathway is essential for a number of biological processes in mammalian systems, including genomic stability and development (Grewal and Rice, 2004). Methylation of histone H3-Lys9 is a common histone mark generally associated with an inactively transcribed promoter. Ting reported that the small double-stranded RNAs that targeted exclusively to the CDH1 promoter could effectively induce transcriptional repression with chromatin changes characteristic of inactive promoters. They successfully detected dimethylation of Lys9 of histone H3 (H3K9me2) only at the CDH1 promoter in cells treated with dsCDH1-1 (Ting *et al*, 2005). In this study, we detected H3K9me3 after treatment of promoter-targeting siRNA. H3K9me3 was detected adjacent to the target region and we also found significant increase in its levels promoter-targeting siRNA-treated cells, which suggests that histone modification participates in gene silencing of HR-HPV E6 and E7 at the transcriptional level. Several publications also demonstrated the consistency between transcriptional gene silencing and DNA methylation in human cells (Morris *et al*, 2004; Castanotto *et al*, 2005). In our result, we found no methylation in the promoter of E6 and E7 with pyrosequencing. It has been reported that the frequency of promoter methylation is probably not always consistent with the inactivation of the promoter and the expression of downstream genes (Castanotto *et al*, 2005), and the representation of epigenetics inducted by siRNA is various (Morris *et al*, 2004; Castanotto *et al*, 2005). Transcriptional gene silencing in human cancer cells could only be accompanied by histone modification, with absence of DNA methylation (Ting *et al*, 2005). Thus, our findings suggest that promoter DNA methylation may not be a kind of mechanism of transcriptional gene silencing of HPV16 E6/E7 induced by siRNA.

In our study, the decreases in E6/E7 mRNA and protein expression were more than 40 and 30%, respectively. The reduction was similar to the results reported by Castanotto *et al* (2005). The causation that the silencing effects shown are moderate is probably of siRNA synthesised *in vitro* and transfected by liposome in the study. Most of the siRNA transfected into cells remains in the cytoplasm and only a small part enters the nucleus; however, the activity of siRNA to the promoter is takes place in the nucleus. Alternatively, siRNA might only gain access to genomic DNA when the nuclear membrane disappears during cell division.

In conclusions, promoter-targeting siRNA effectively and simultaneously knocks down both extraneous viral genes HPV 16 E6 and E7 at the transcriptional level, and consequently inhibits proliferation and induces death in HPV 16-infected cells. This transcriptional repression is probably induced by histone modification rather than by an alteration of DNA methylation.

## Figures and Tables

**Table 1:** The primer sequences for PCRs

**Primer**	**Sequence**
E6F	5′-TCAAAAGCCACTGTGTCCTG-3′
E6R	5′-CGTGTTCTTGATGATCTGCA-3′
E7F	5′-ATTAAATGACAGCTCAGAGGA-3′
E7R	5′-GCTTTGTACGCACAACCGAAGC-3′
*β*-ActinF	5′-TTCCAGCCTTCCTTCCTGG-3′
*β*-ActinR	5′-TTGCGCTCAGGAGGAGCAAT-3′
msp7708F	5′-TTTTGGTTTGTTTTAATTAA-3′
msp161R	5′-ACAACTCTATACATAACTATAATA-3′
msp115R	5′-ATCCTAAAACATTACAATTCTCTTTTAATA-3′
msp53mR	5′-CGATTCAACCGATTTCGATTACGCCCTT-3′
msp53nmR	5′-CAATTCAACCAATTTCAATTACACCCTT-3′
Pyro-msp11F	5′-TTTATGTATAAAATTAAGGG-3′
F14	5′-ATGTATAAAACTAAGGGCGTAA-3′
R113	5′-CCTGAAACATTGCAGTTCTC-3′
GAPDH-F	5′-TACTAGCGGTTTTACGGGCG-3′
GAPDH-R	5′-TCGAACAGGAGGAGCAGAGAGCGA-3′

Abbreviations: GAPDH=glyceraldehyde-3-phosphate dehydrogenase; PCR=polymerase chain reaction.
